# Do Domain Knowledge and Retrieval Practice Predict Students’ Study Order Decisions?

**DOI:** 10.3390/jintelligence10040122

**Published:** 2022-12-09

**Authors:** Addison L. Babineau, Amber E. Witherby, Robert Ariel, Michael A. Pelch, Sarah K. Tauber

**Affiliations:** 1 Department of Psychology, Texas Christian University, Fort Worth, TX 76129, USA; 2 Department of Psychology, Creighton University, Omaha, NE 68178, USA; 3 Department of Psychology, Virginia Wesleyan University, Virginia Beach, VA 23455, USA; 4 School of Environment, Washington State University, Pullman, WA 99164, USA

**Keywords:** study order decisions, interleaved vs. blocked study, prior knowledge, retrieval practice

## Abstract

Learning complex concepts is necessary for student success, but it is often challenging. Learning such concepts can be influenced by students’ study order choices during learning to switch to a new category (interleaved study order) or stay within the same category (blocked study order). Students often prefer stay decisions during learning and make relatively few switch decisions; however, an open question is whether students’ switch decisions are related to their level of prior knowledge in the domain and the learning strategy they use (retrieval practice versus study). To examine these relationships, we recruited undergraduate students from an introductory geology course. Prior to the course modules on rock classification, students self-rated their knowledge, took a prior knowledge test, classified rock exemplars by completing study or retrieval practice trials, and made study order choices. Students then completed assignments and attended lectures in their geology course on igneous, sedimentary, and metamorphic rocks. Next, students self-rated their knowledge, took a new prior knowledge test, completed study or retrieval practice trials, made study order decisions, took final classification tests, and self-reported beliefs about study order choices. Even though students’ knowledge increased after course modules on rock identification, and most students believed that domain knowledge impacts study decisions, prior knowledge did not predict students’ switch decisions. In contrast, students who completed retrieval practice trials made substantially more switch decisions (i.e., interleaved study) than did students who completed study trials.

## 1. Introduction

Imagine taking an introductory geology class as a college student. A portion of the course emphasizes classifying distinct categories of rocks based on their origin because this knowledge is foundational for the field. Learning such complex and naturally occurring concepts is challenging, and students can struggle to determine the best strategies to implement to support their learning. Most important for the current research, how exemplars are ordered during study (blocked or interleaved) can impact students’ learning (e.g., Carvalho and Goldstone 2014; Kornell and Bjork 2008), but students do not always make optimal study order decisions (e.g., Kornell and Bjork 2008; Tauber et al. 2013). As such, our goals were to evaluate the degree to which prior knowledge in the domain and retrieval practice trials impact students’ study order choices.

During category learning, concepts can be arranged so that exemplars from different categories are studied in succession; this is referred to as interleaved study order. For instance, geology students can prepare for class by studying an example igneous rock followed by a metamorphic rock and then a sedimentary rock. By contrast, blocked study order involves studying multiple exemplars from the same category consecutively. To illustrate, geology students could study an example igneous rock followed by two new examples of igneous rocks. Relative to blocked study order, interleaved order often better supports students’ category learning (e.g., Eglington and Kang 2017; Kang and Pashler 2012; Kornell and Bjork 2008; Rohrer and Taylor 2007; Samani and Pan 2021; Yan et al. 2016; Zulkiply et al. 2012; Yan and Sana 2021; Yan et al. 2020; for meta-analyses, see Brunmair and Richter 2019; Firth et al. 2021). However, improvements in learning with interleaved study order are not always observed (e.g., Abel et al. 2021; Carvalho and Goldstone 2014; Elio and Anderson 1981; Lu et al. 2020; Shah et al. 2016; for moderators, see Brunmair and Richter 2019; Firth et al. 2021) and may depend on characteristics of the learner.

The most beneficial study order during category learning can be related to students’ level of prior knowledge in the domain (Guo and Pang 2011; Guo et al. 2014; Rittle-Johnson et al. 2009; but see Quilici and Mayer 1996). Research on learning mathematics indicates that, with low domain knowledge, students’ learning has benefited from blocked study order or an order that begins with blocked study and gradually reduces it (Rau et al. 2010), and by studying similar examples (Braithwaite and Goldstone 2015). By contrast, with increased domain knowledge, varied examples during learning appears to be beneficial and there are minimal benefits from blocked study (Braithwaite and Goldstone 2012; 2015; Rau et al. 2010; but see Osana et al. 2017). Importantly, in these experiments, study order was experimenter-controlled, and no research to-date has evaluated whether students’ prior knowledge in the domain is related to their study order choices made during self-regulated learning.

During self-regulated learning, students make decisions about how to study new information. To make interleaved study decisions, students choose to switch between categories (i.e., study a different type of rock next). The alternative to switch decisions are decisions to stay within the same category (i.e., study the same type of rock next), which leads to blocked study order. Prior research has established that most students believe blocked study is more beneficial for their learning than is interleaved study (Kornell and Bjork 2008; Morehead et al. 2016; McCabe 2012; Yan et al. 2016) or at least that some amount of blocked study should be included during their learning (Yan et al. 2017). Consistent with these subjective reports, students frequently choose to block their study by staying within a category and minimizing switch decisions (Lu et al. 2020; Tauber et al. 2013). Because students’ preference to block has been established, our goal was to explore when and why students deviate from blocked study by choosing to interleave. Thus, our primary measure of interest was students’ switch decisions (cf. Babineau and Tauber, forthcoming; Lu et al. 2020).

We explored the novel possibilities that students’ study choices are related to their prior knowledge in the domain of interest and are influenced by engaging in retrieval practice (e.g., self-testing) during learning. Retrieval practice is a highly effective learning strategy that has broad educational applicability (Carpenter et al. 2022; Roediger and Karpicke 2006). Research shows retrieving domain-relevant information from memory opposed to simply restudying it can improve student learning in biology (Pan et al. 2016; Pan et al. 2018), chemical engineering (Darabi et al. 2007), foreign language courses (Kang et al. 2013; Metcalfe et al. 2007), history (Carpenter et al. 2009; Pan et al. 2016), mathematics (Kang et al. 2011), and psychology (Foss and Pirozzolo 2017). Retrieval practice can even facilitate learning to classify exemplars of natural categories such as birds (Jacoby et al. 2010). Thus, it may be an ideal tool for learning to classify rocks in introductory geology courses.

Concerning students’ study choices, retrieval practice provides metacognitive benefits that typically improve the quality of students’ self-regulated decisions during learning (Agarwal et al. 2008; Ariel et al. 2021; Little and McDaniel 2014; Tullis et al. 2013). When students successfully retrieve information from memory, it signals to the student that they know the material and should focus on information that is less likely to be recalled. Consequently, students typically terminate the study of material after they have successfully recalled it (Dunlosky and Rawson 2015; Karpicke 2009; Kornell and Son 2009). Thus, it follows that students who engage in retrieval practice should prefer to stop studying exemplars of a particular category and switch to a new category following successful retrieval practice trials.

As previously noted, students with existing prior knowledge have benefitted from variation in examples and less from blocked study (e.g., Braithwaite and Goldstone 2012; 2015; Rau et al. 2010); thus, ideally, switch choices should be associated with increased prior knowledge in the domain. Furthermore, when students have accurate foundational knowledge on which to build new learning, they are likely to answer a greater proportion of practice trials correctly during category learning. This may lead students to conclude that they have learned the category well enough to move on, i.e., to switch to a new category. By contrast, students with low prior knowledge have benefitted from blocked study (e.g., Braithwaite and Goldstone 2012; 2015; Rau et al. 2010; Rittle-Johnson et al. 2009); therefore, few switch choices associated with lower prior knowledge is preferrable. With little-to-no prior knowledge, students are likely to incorrectly answer many practice trials. Students may interpret such failures as representative of incomplete knowledge about the category and decide to revisit it to learn it better. In other words, switch decisions would be reduced by students who have low prior knowledge.

The relationship between prior knowledge and students’ switch decisions during self-regulated learning is unknown. Thus, we measured students’ prior knowledge with self-assessments (Braithwaite and Goldstone 2015; Große and Renkl 2007) and objective tests (Braithwaite and Goldstone 2012; Große and Renkl 2007; Rau et al. 2010) prior to completing two phases of a self-regulated learning task. Phase 1 occurred prior to covering the target content in the course and Phase 2 occurred after covering this content, which allowed us to investigate how changes in prior knowledge affect switch decisions. Further, it is an open question as to the impact of retrieval practice on students’ switch decisions. To evaluate this, some students were required to complete practice classification trials whereas others were not. We conducted the reported experiment by using authentic classroom materials and in conjunction with students’ course activities for introductory college courses in geology. In these classes, students studied how to classify rocks into respective categories at the basic level (igneous, metamorphic, or sedimentary), which they learned during the experiment as well.

## 2. Materials and Methods

### 2.1. Participants

We pre-registered our data collection plans, experimental design, and guidelines for analyses (visit https://osf.io/za328/?view_only=52e47042b66c4b4cb151dd7d99e9f57e for more information). We recruited students from an introductory geology course and planned to recruit 80–100 students based on typical enrollment in that course. During the fall term, 9 students were recruited; during the spring term, 67 students were recruited. Of the 76 recruited students, 4 students did not return for Phase 2 of the experiment, 4 students did not attend both geology labs that occurred between the phases of the experiment, and 2 students’ data were lost due to a research error. Thus, 66 students were included in the sample and randomly assigned to the study group (*n* = 33) or retrieval practice group (*n* = 33). Most students were college-aged (study group, *M* = 19.73 years, *SE* = .99; retrieval practice group, *M* = 19.03 years, *SE* = .14; one student did not report age) and identified as women (study group, 66.7%, *n* = 22, 11 men; retrieval practice group, 78.8% *n* = 26, 5 men, 1 gender diverse, and 1 woman and gender diverse) and White (study group, 63.7%, *n* = 21, 4 White and Latino, 3 Latino, 3 Asian, 1 Black and Latino, and 1 student did not report ethnicity; retrieval practice group, 69.7%, *n* = 23, 4 White and Latino, 2 White and Native American, 1 Black, 1 Latino, 1 White and Black, and 1 White and Pacific Islander). The groups did not significantly differ in age, *t*(63) = .69, *p* = .494, gender, *χ*^2^ (2) = 4.58, *p* = .10, or ethnicity, *χ*^2^ (10) = 12.2, *p* = .27.

Students’ reports of their prior experience with geology can be found in Table 1. Few students reported having majors or hobbies related to geology. Further, there were no significant differences in prior geology experience between the groups, *χ*^2^s ≤ .73, *p*s ≥ .392.

### 2.2. Geology Course Description

Understanding the Earth is a four-credit introductory geology course consisting of seven content modules and ten-to-eleven laboratory activities (the number of laboratory activities is dependent upon the semester). There are 3–4 lecture sections with 100–175 students enrolled in each. There are 14–18 lab sections with up to 25 students enrolled in each. Lecture sections are associated with 4–7 lab sections. This is designed so that students will attend lab with only students from the same lecture section. Lectures occurred twice per week for 50 min, and labs occurred once per week for 150 min. Lectures occurred in classrooms with fixed rows of seats in an auditorium-style layout, and labs were in smaller rooms in which groups of 3–4 students sit together at a single table.

The course content was aligned with three course objectives: (1) synthesize the connections between human civilization, the geosphere, and the hydrosphere; (2) elaborate on how Earth’s internal and surface processes shape the geosphere over geologic timescales; and (3) evaluate the cultural and social aspects of natural resources. Lectures comprised a mixture of traditional content delivery, in-class response questions, and small-group work. The course content that was most relevant to the current study was the “Rocks of the U.S.” lecture module, which occurred after Phase 1 and before Phase 2 of the experiment. This module covered the learning goals pertaining to igneous, metamorphic, and sedimentary rocks. During this portion of the course, students learned about the physical and chemical characteristics of the major rock types, how geologists use those characteristics to identify different rock types, and how geologists use rock types to interpret geologic processes that occur in the lithosphere and in deeper parts of the Earth. The professor’s primary goal was for students to learn to distinguish between the three basic-level categories of rocks (igneous, metamorphic, and sedimentary).

Laboratory activities were facilitated by a graduate teaching assistant and consisted of group work during which students made observations, collected data, and applied concepts to scaffolded experiments to explore geologic phenomena. The lab on igneous and metamorphic rocks began with a student-driven exploration about how cooling rate and mineral composition alters various types of igneous rocks. Students were then given a selection of unknown igneous rocks to classify. Next, during the portion of the lab covering metamorphic rocks, students explored the various ways in which changes in pressure, temperature, and fluids alter rocks to create the unique textures and physical characteristics associated with metamorphism of earth materials. After exploring these characteristics, students classified an unknown selection of commonly occurring metamorphic rocks. The sedimentary rocks lab covered the physical and chemical properties of different sedimentary rocks derived from the weathering and erosion of other rock types, and of those that form from chemical processes such as the evaporation of seawater. During the lab, students explored physical processes of sedimentary rocks formed from weathering and erosion such as grain size, grain sorting, grain shape, and minerology. Next, students classified the physical characteristics of sedimentary rocks that form due to the evaporation of fresh and saline water, and biochemical processes such as the formation of coal and limestone. Finally, students were presented with a sample of 14 unknown commonly occurring sedimentary rock samples that they worked together to classify.

Performance on the igneous, metamorphic, and sedimentary lab activities (both of which occurred after Phase 1 and before Phase 2 of the experiment) is directly relevant to the current study (see Table 2). Furthermore, each lecture was preceded by an online quiz due the evening before and that was related to content that would be explored by students during the upcoming class period. Students took two pre-class quizzes on igneous, metamorphic, and sedimentary rocks after completing Phase 1 and prior to Phase 2 of the experiment. Students also took three exams during the semester, and questions about igneous, metamorphic, and sedimentary rocks were included on the mid-term (which occurred between Phase 1 and Phase 2 of the experiment) and the final exam (which occurred after Phase 2 was completed). Exams were mostly multiple choice with one written response question. In sum, the course activities that are most relevant to the current study are students’ performance on the two lab assignments on igneous, metamorphic, and sedimentary rocks, two pre-class quizzes on igneous, metamorphic, and sedimentary rocks, the mid-term exam, and the final exam.

Course performance data were obtained for students enrolled in the spring 2022 semester, but not for the 9 students enrolled during the fall 2021 semester; however, it should be noted that students from the fall comprised a relatively small portion of the total sample (12%). As evident from Table 2, students performed well on pre-class quizzes and laboratory assignments on igneous, metamorphic, and sedimentary rocks. Students scored an average of a B on the mid-term exam, and an average of a C on the final exam. Most important, the groups did not significantly differ in their performance on the pre-class quiz on igneous and metamorphic rocks, the pre-class quiz on sedimentary rocks, the laboratory assignment on igneous and metamorphic rocks, the laboratory assignment on sedimentary rocks, the mid-term exam, or the final exam, *t*s < 1.88, *p*s > .067. There was only one significant difference between groups. Even though both groups performed well and ceiling-levels of performance were observed, the retrieval practice group performed better on the igneous and metamorphic rocks laboratory assignment than did the study group, *t*(56) = 2.39, *p* = .02, *d* = .63.

### 2.3. Materials

Students learned to classify rocks into three rock categories (igneous, metamorphic, or sedimentary). Rock categories have a hierarchical structure; thus, each category was composed of 4 subcategories: Igneous—peridotite, pegmatite, granite, and gabbro; Metamorphic—gneiss, marble, phyllite, and schist; Sedimentary—rock salt, shale, chert, and bituminous coal. Rock subcategories were previously normed on the dimensions of family-resemblance and distinctiveness by Nosofsky et al. (2018). Using these dimensions, several normative analyses were conducted to ensure that the basic categories are not significantly different from each other. Specifically, each basic category had the same degree of similarity between exemplars, *F*(2, 9) = .65, *p* = .547, as well as the same degree of similarity between categories, *F* (2, 9) = .80, *p* = .480 (i.e., one category was not more distinct than another). Importantly, each rock subcategory was included in activities that took place in the Understanding the Earth labs; thus, each rock category used in the experiment received direct instruction in the classroom after Phase 1 and before Phase 2 of the experiment.

Each category was composed of 48 total exemplars (12 exemplars from each subcategory). From each category, 4 exemplars were randomly selected (1 from each subcategory) for the Phase 1 prior knowledge test, 4 exemplars were randomly selected (1 from each subcategory) for the Phase 2 prior knowledge test, 12 exemplars were randomly selected (3 from each subcategory) for the novel classification test, and the remaining 28 exemplars (7 from each subcategory) were used during the self-regulated learning phase and studied exemplar classification tests. In total, 12 exemplars were used for the Phase 1 prior knowledge test, 12 exemplars were used for the Phase 2 prior knowledge test, 36 exemplars were used for the novel classification test, and 84 exemplars were used for the self-regulated learning phase and studied classification test. Different exemplars were used for each task (i.e., Phase 1 prior knowledge test, Phase 2 prior knowledge test, and novel classification test) with the exception that the same exemplars were used for the self-regulated learning phase and the studied exemplar classification test. The exemplars assigned to each group were the same for each student and across both time periods.

### 2.4. Procedure

#### 2.4.1. Phase 1

**Figure 1:**
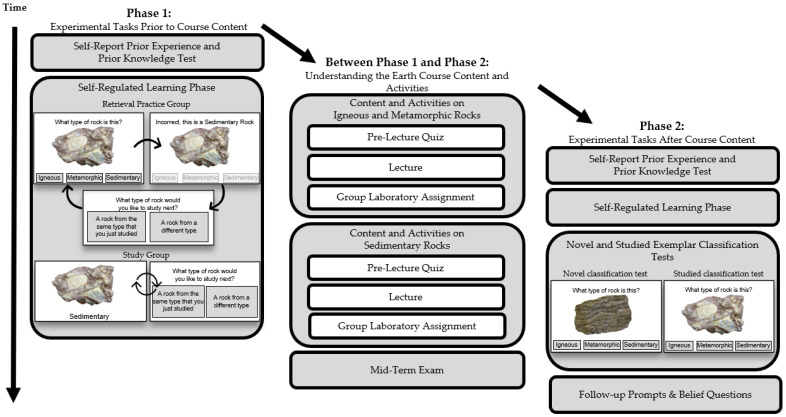
Overview of the experiment tasks and timeline. During the self-regulated learning phase, participants were randomly assigned to the retrieval practice group or study group. Group assignment was maintained during the Phase 2 self-regulated learning phase.

An overview of the experimental phases is shown in Figure 1. Phase 1 of the experiment took place during the two weeks prior to the course content on igneous, sedimentary, and metamorphic rocks from lectures and labs for the Understanding the Earth course. Students began Phase 1 of the experiment by completing 4 yes/no questions reporting their prior experience with geology (see Table 1) and rating their prior knowledge of identifying rocks by responding to the question, *“What is your own rated level of expertise at identifying rocks?”,* on a scale from 1 to 7. Then, students completed the Phase 1 prior knowledge test. During this test, students classified 12 rock exemplars as igneous, metamorphic, or sedimentary rocks. The exemplars were shown on the screen one-at-a-time, with the three category names displayed on buttons beneath the exemplar in alphabetical order. Below the 3 rock classification buttons, there was an “I don’t know” button. Students were encouraged to select the “I don’t know” button if they were unsure of the correct rock classification. Students did not receive feedback during the Phase 1 prior knowledge test, and the program automatically proceeded to the next exemplar after the student selected their answer. The Phase 1 prior knowledge test was self-paced, and the exemplars were shown in a random order for each student.

Following the Phase 1 prior knowledge test, students proceeded to the self-regulated learning phase. Students began the self-regulated learning phase with a randomly selected exemplar. For students in the study group, the exemplar was displayed in the center of the screen with the correct category name shown beneath it. The exemplar remained on the screen for 3 s, then a “next” button appeared in the lower right-hand corner of the screen. Students could view the exemplar for as long as they wanted prior to selecting the “next” button.

For students in the retrieval practice group, the exemplar was shown in the center of the screen with the question, “*What type of rock is this?*”, shown above it. Below the exemplar, the three category names were displayed on buttons in alphabetical order. Students had unlimited time to classify the exemplar and did so by clicking on the button with the corresponding category name. An “I don’t know” option was not provided during the self-regulated learning phase. After classifying the exemplar, students received corrective feedback on the trial. When students correctly classified the exemplar, “*Correct, this is a [correct category name]*” was displayed above the exemplar for 3 s. When students incorrectly classified the exemplar, “*Incorrect, this is a [correct category name]*” was displayed above the exemplar for 3 s. After viewing the feedback, a “next” button appeared in the lower right-hand corner of the screen. Students could view the feedback for as long as they wanted prior selecting the “next” button.

After completing a self-regulated trial and selecting the “next” button, students in both groups made a study order selection. Specifically, students responded to, “*What type of rock do you want to study next?*” by selecting 1 of 2 buttons beneath it that were labelled, “*A rock from the same type that you just studied*” and “*A rock from a different type*”. The button location was counterbalanced between students. When students selected to study the same type of rock again, a different exemplar from the same category as the previous trial was randomly selected and shown on the following trial. For example, if a student completed a trial with an igneous exemplar and then selected to study the same rock category again, the following trial would display a different exemplar of an igneous rock. When students selected to study a different rock category, an exemplar from a different rock category was randomly selected and shown on the following trial. For example, if a student completed a trial with an igneous exemplar and then selected to study a different rock category, the following trial would display an exemplar from a different category, such as an exemplar of a metamorphic rock. Students were allowed to study each rock category as many times as desired. If a student selected to study a rock category in which they had previously studied all 28 of the study exemplars, then the exemplars were repeated on subsequent trials; that is, students could select to study all 84 study exemplars, and they could study them more than once. All aspects of study selection were self-paced.

To obtain an adequate number of observations, students in both groups proceeded through the self-regulated learning phase until they had completed at least 40 self-regulated trials (i.e., they studied or practiced classifying at least 40 exemplars and made the subsequent study order selection for each). After 40 trials, an option to terminate study was provided. Even so, at the outset of the study phase, students were instructed to study as many exemplars as desired and for as long as they wanted. Students were encouraged to only end the study phase when they had learned the rock categories well enough that they would no longer need to guess on a future test. Upon selecting to terminate the study phase, Phase 1 was concluded, and students were directed to schedule their session for Phase 2.

#### 2.4.2. Phase 2

Phase 2 of the experiment took place after the course content, lectures, mid-term exam, and two rock classification labs in the Understanding the Earth course. Phase 2 of the experiment began with 2 yes/no questions reporting prior experience with geology (see Table 1) and completing the same measure as used at the start of Phase 1 for self-rating their prior knowledge of identifying rocks. Next, students completed the Phase 2 prior knowledge test. The procedure for the Phase 2 prior knowledge test was identical to that of the Phase 1 prior knowledge test; however, the 12 exemplars were different from those selected for the Phase 1 prior knowledge test. Following the Phase 2 prior knowledge test, students completed a self-regulated learning phase, which was identical to the self-regulated phase they completed during Phase 1.

After finishing the self-regulated learning phase, students began the final test phase. During the test phase, students completed a novel classification test and a studied exemplar classification test, with the order of the tests counterbalanced between students. During the novel classification test, students classified 36 exemplars that had never been seen from the 3 rock categories they studied. Beneath each exemplar, the 3 rock categories were listed alphabetically and in a fixed order for each trial and student. Students classified each of the 36 novel exemplars, one-at-a-time, by selecting the button for the rock category. During the test, exemplar order was randomized for each student. The test was self-paced, and no feedback was provided. The procedure for the studied exemplar classification test was identical to the novel classification test; however, students classified the 84 exemplars they saw during the self-regulated learning phase. An “I don’t know” option was not provided on either test.

Following the test phase, students were presented with two prompts aimed at understanding why they made certain study decisions (i.e., “*When I selected to study the same type of rock, I did so because”* and “*When I selected to study a different type of rock, I did so because*”; see Table 3). They were provided with a series of options as well as a free-response box to enter their answers. The options were designed to represent a variety of reasons for why a student may select to switch or stay (cf. Babineau and Tauber, forthcoming), and we also provided the free-response box to ensure that students could report their experience if the provided options did not represent them. The prompts were displayed one-at-a-time and the order of the prompts was counterbalanced between students. The options were displayed below each prompt and in a random order for each student. The prompts were self-paced, and students could select as many or as few options as they wanted. An attention check was included within the options associated with the prompt about switching study decisions. For the attention check, students selected the option to show they were paying attention. Most (65.5%) students correctly selected the attention check indicating that they read each of the options before submitting their answers.

Following the study decision prompts, students were asked about their beliefs about study choices and expertise. Students were given the following instructions, *“Imagine that two new people completed the same task as you. Just like you, they were given examples of rocks to study for an upcoming test. After studying each rock example, they decided what they wanted to study next—a rock from the same type or a rock from a different type.”* Next, some students read, *“One person has a lot of knowledge in Geology—in fact, they’re an expert. This person has been studying geology for years and has taken courses on rock classification. The other person has no knowledge in geology. This person has never taken a course on geology or rock classification. What types of study decisions do you think these two people would make?”* Other students read the identical information but information about the novice was presented first (i.e., the order was counterbalanced order across students). Students then responded to the question, *“Do you think that these two people would make the same study choices or different study choices?”* The order of same and different study choice options in this question was counterbalanced across students. Students who reported that the expert and the novice would make different study order decisions were asked how they believed an expert in geology would select to study, as well as how a novice in geology would select to study (see Table 4). These follow-up questions were each self-paced and the order was counterbalanced between students. Following the post-experiment questions, students were debriefed and compensated. Students who completed the experiment during fall 2021 were compensated with $20 in gift cards. Students who completed the experiment during spring 2022 were compensated with extra credit in their Understanding the Earth course.

## 3. Results

To examine the impact of prior knowledge on students’ study order decisions, we first report students’ self-rated expertise in identifying rocks and their prior knowledge scores from both phases of the study. Then, we report omnibus analyses and mixed-effects models (MEM) exploring students’ study order decisions. Next, we report omnibus analyses of the retrieval practice group’s performance during the self-regulated learning phases. Then, we report omnibus and MEM analyses exploring students’ studied exemplar and novel test performance. Finally, we report the outcomes of the follow-up questions about students’ rationale for their study choices.

### 3.1. Changes in Prior Knowledge

#### 3.1.1. Students’ Subjective Reports of Prior Knowledge

To examine students’ subjective assessments of their ability to identify rocks, they self-rated their expertise on a 7-point scale (i.e., 1 = novice, 7 = expert) at the start of Phase 1 and again at the start of Phase 2. Students’ ratings significantly increased from Phase 1 to Phase 2 in the study group (Phase 1: M = 1.5, SE = .12; Phase 2: M = 2.33, SE = .12) and the retrieval practice group (Phase 1: M = 1.5, SE = .12; Phase 2: M = 2.64, SE = .11), F(1, 64) = 56.40, *p* < .001, ɳ^2^ = .215. There was no significant difference between the groups in their self-rated expertise with identifying rocks, F(1, 64) = .64, *p* = .428, nor was there a significant interaction between group and phase, F(1, 64) = 1.38 *p* = .245. Thus, both groups judged that their ability to identify rocks improved after course content and assignments on igneous, metamorphic, and sedimentary rocks.

#### 3.1.2. Objective Assessments of Students’ Prior Knowledge

To obtain an objective measure of students’ prior knowledge, prior knowledge scores were calculated independently for each student by dividing the number of correct responses on the prior knowledge tests by the total number of trials. Students’ “I don’t know” responses were treated as errors of omission and were scored as zeros. Of most interest, a mixed-effects analysis of variance (ANOVA) indicated that students’ prior knowledge scores significantly increased from Phase 1 (M = .16, SE = .02) to Phase 2 (M = .32, SE = .02), F(1, 64) = 35.15, *p* < .001, ɳ^2^ = .173. Students’ prior knowledge scores did not significantly differ between groups, F(1, 64) = .12, *p* = .73, nor was there a significant interaction with phase and group, F(1, 64) = .19, *p* = .66. Further, students’ use of the “I don’t know” response significantly declined from Phase 1 (M = 4.56, SE = .52) to Phase 2 (M = 1.39, SE = .29, t(65) = 7.07, *p* < .001, d = .87, which indicates that not only did students’ knowledge increase, students attempted to answer more prior knowledge questions at Phase 2 as well.

In sum, students had different levels of knowledge about identifying igneous, metamorphic, and sedimentary rocks at Phase 2 relative to Phase 1. Students knew more about identifying rocks following the rock identification units in their geology course (Phase 2) compared to the little knowledge students had about rock identification prior to those units (Phase 1). These objective prior knowledge scores were consistent with students’ self-ratings of their knowledge with identifying rocks. Because self-ratings are not direct measures of actual knowledge and the self-ratings were broad (i.e., general ability to identify rocks), in the subsequent analyses we used the objective measures of prior knowledge, which directly evaluated students’ knowledge of igneous, sedimentary, and metamorphic rocks.

### 3.2. Students’ Study Order Decisions

During the self-regulated learning phase, students in both groups were required to complete 40 trials, and they could persist beyond 40 by completing as many more trials as desired. Thus, we assessed the number of trials completed for each student and each phase by summing the total number of switch and stay trials. A mixed-effects ANOVA revealed that the number of trials completed during self-regulated learning did not significantly differ between the study group (M = 44.70, SE = 1.21) and the retrieval practice group (M = 44.27, SE = 1.14), F(1,64) = .05, *p* = .83, nor did the number of trials completed significantly differ between Phase 1 (M = 44.27, SE = 1.17) and Phase 2 (M = 44.70, SE = 1.18), F(1,64) = .10, *p* = .75. Even though there was a significant interaction between group and phase, F(1, 64) = 4.84, *p* = .031, ɳ^2^ = .03, post hoc comparisons with a Bonferroni correction did not reach the threshold for statistical significance, ts < 1.69, ps > .10. The interaction was driven by a slight increase in completed trials from Phase 1 (M = 42.58, SE = 1.03) to Phase 2 (M = 44.97, SE = 2.01) by the retrieval practice group whereas a slight decrease in completed trials from Phase 1 (M = 45.97, SE = 2.09) to Phase 2 (M = 43.42, SE = 1.21) was observed for the study group.

Further, because the self-regulated learning phase was self-paced, we assessed the time spent during self-regulated learning for each student by summing the total number of seconds spent studying or completing retrieval practice trials, viewing feedback, and making study order decisions. A mixed-effects ANOVA revealed that students spent significantly more time on self-regulated learning during Phase 1 (M = 296.08 s, SE = 15.09 s) compared to Phase 2 (M = 276.89 s, SE = 16.19 s), F(1,64) = 4.13, *p* = .046, ɳ^2^ = .01. However, time spent during self-regulated learning did not significantly differ between the study group (M = 262.81 s, SE = 20.06 s) and the retrieval practice group (M = 310.15 s, SE = 8.54 s), F(1,64) = 2.59, *p* = .11, nor was the interaction significant, F(1,64) = 2.46, *p* = .12.

**Figure 2:**
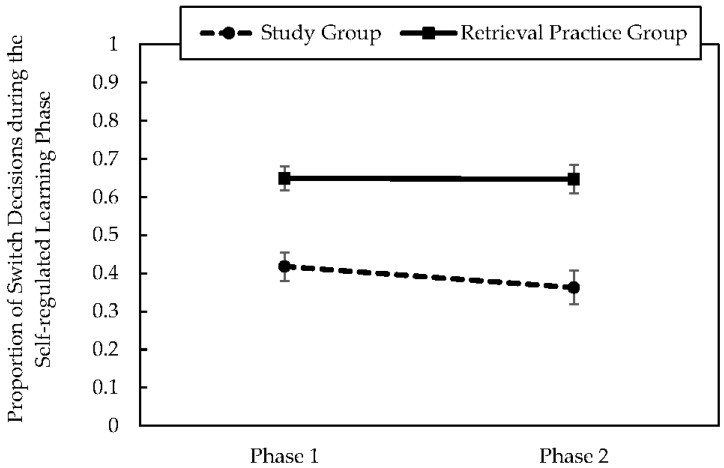
Values represent the mean proportion of switch decisions during each of the self-regulated learning phases for each group. Error bars represent the standard error of the mean.

To evaluate students’ study order decisions, we focused on the frequency with which students chose to switch (i.e., interleave) during learning. To do so, we computed the proportion of study trials during each phase for which students made the decision to switch (i.e., study an item from a different category). As shown in Figure 2, students in the retrieval practice group made switch decisions more often compared to students in the study group. Moreover, students’ study decisions remained largely unchanged from Phase 1 to Phase 2. These outcomes were confirmed with a 2 (group: study group, retrieval practice group) × 2 (phase: Phase 1, Phase 2) mixed ANOVA on students’ proportion of switch decisions. The main effect of group demonstrated that students in the retrieval practice group (M = .66, SE = .03) chose to switch on a greater proportion of trials compared to students in the study group (M = .40, SE = .03), F(1, 64) = 31.90, *p* < .001, ɳ^2^ = .27. The main effect of phase was not significant, F = 1.09, *p* = .30, nor was the interaction between group and phase, F < 1, *p* = .35. These analyses provide information about group differences, but not about students’ study decisions on each trial.

**Figure 3:**
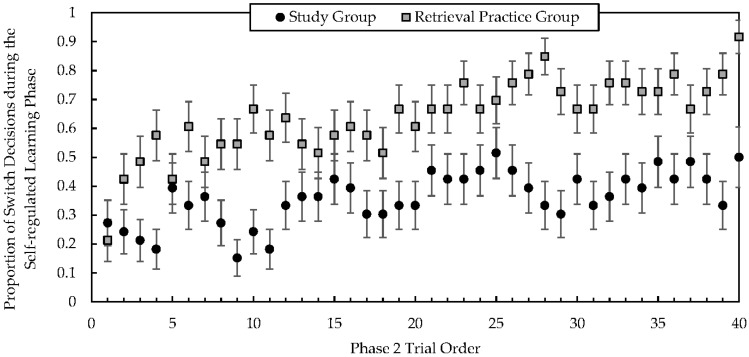
Values represent the mean proportion of switch decisions for each trial order position of the Phase 2 self-regulated learning phase separately for each group. Even though students could select to complete more than 40 trials, all students were required to complete 40 trials before proceeding to the test phase; thus, the graph presents study order decisions from trial 1 to trial 40. Error bars represent the standard error of the mean.

To investigate students’ study decisions on each trial during Phase 2, we conducted MEM analyses exploring the relationship between changes in students’ prior knowledge and item-level study decisions. We focused on students’ decisions during Phase 2 because a primary goal was to explore how students’ decisions related to changes in their prior knowledge from Phase 1 to Phase 2. Further, MEM analyses are ideal for analyzing nested data (i.e., items nested within students) because they control for characteristics of the students and items with random coefficients, and they account for non-independence of data (e.g., Middlebrooks and Castel 2018; Middlebrooks et al. 2016). A logistic MEM with random participant effects was used to predict students’ study decision on each trial (0 = stay, 1 = switch). Given that our focus was on students’ decision to stay or switch on the next study trial, we removed go-to-test trials for this analysis. Condition (0 = study group, 1 = retrieval practice group), group-centered change in prior knowledge from Phase 1 to Phase 2 (i.e., each student’s Phase 2 prior knowledge score minus their Phase 1 prior knowledge score), the interaction between condition and prior knowledge change, and trial order were entered as predictors. Trial order was entered as a random effect. Consistent with the ANOVA outcomes, students in the retrieval practice group were 3.06 times more likely to switch compared to students in the study group, b = 1.12 (SE = .39), t = 2.90, *p* = .004, CI [.36, 1.88]. Trial order was also a significant predictor, b = .06 (SE = .01), t = 6.72, *p* < .001, CI [.04, .07], indicating that students were 1.06 times more likely to switch as they completed more trials (see Figure 3). Change in prior knowledge was not a significant predictor, t = 1.40, *p* = .16, nor was the interaction between condition and prior knowledge change, t < 1, *p* = .82.

We conducted a similar MEM for the retrieval practice group only to explore whether their performance during practice classification influenced their likelihood of switching during Phase 2. A logistic MEM with random participant effects was used to predict students’ study decisions on each trial (0 = stay, 1 = switch). Students’ performance on each practice classification trial (0 = incorrect, 1 = correct), change in prior knowledge (group-centered), the interaction between practice classification performance and prior knowledge change, and trial order were entered as predictors. Trial order was entered as a random effect. A significant effect of practice classification performance, b = .42 (SE = .15), t = 2.72, *p* = .006, CI [.12, .72], indicated that students were 1.52 times more likely to switch after getting an item correct relative to incorrect. Trial order was also a significant predictor, b = .08 (SE = .02), t = 5.17, *p* < .001, CI [.05, .11], indicating that students were 1.08 times more likely to switch as they completed more trials. Prior knowledge change was not a significant predictor, t < 1, *p* = .61, nor was the interaction between practice classification performance and prior knowledge change, t = 1.89, *p* = .06.

### 3.3. Practice Classification Performance during the Self-Regulated Learning Phases

For students in the retrieval practice group, performance during self-regulated learning at Phase 1 and Phase 2 was calculated independently for each student. For each phase, the number of correct practice classification trials during self-regulated learning was divided by the total number of trials completed. Overall, students performed well during self-regulated learning for both Phase 1 and Phase 2. There were no significant differences between students’ practice classification performance during Phase 1 (M = .70, SE = .02) and Phase 2 (M = .72, SE = .03), t(23) = .91, *p* = .37. Thus, students’ retrieval practice performance was consistent across the two phases of the experiment.

### 3.4. Performance on the Studied Exemplar Test

There was no difference in studied exemplar test performance between the study group (M = .56, SE = .02) and the retrieval practice groups, (M = .60, SE = .02), t = 1.15, *p* = .25. To evaluate the impact of prior knowledge on students’ item-level studied exemplar test performance (0 = incorrect, 1 = correct), we conducted logistic MEMs with random participant effects.

In the first analysis including both groups, and we entered condition (0 = study group, 1 = retrieval practice group), change in prior knowledge (group-centered), the total number of times that specific exemplar was studied, the proportion of trials that the student chose to switch after studying that exemplar, and the interaction between condition and prior knowledge change to predict students’ studied exemplar performance. The total number of times each exemplar was studied was the only significant predictor, b = .26 (SE = .07), t = 3.98, *p* < .001, CI [.13, .39]. With each additional time a student studied a specific exemplar during self-regulated learning, they were 1.30 times more likely to get that exemplar correct on the studied exemplar test. The remaining predictors and the interaction were not significant, ts < 1.67, ps > .09.

In the second analysis, we explored whether students’ practice classification performance in the retrieval practice group impacted their studied exemplar test performance. To do so, a logistic MEM was used to predict students’ studied exemplar performance at the item level (0 = incorrect, 1 = correct). Change in prior knowledge (group-centered), the total number of times the exemplar was studied, the proportion of trials that the student chose to switch after studying that exemplar, and the total proportion of trials for which students correctly classified that exemplar were entered as predictors. Similar to the previous analysis, students were 1.36 times more likely to get a studied exemplar correct with more study trials during self-regulated learning, b = .31 (SE = .10), t = 3.20, *p* = .001, CI [.12, .49]. Students’ practice classification performance during self-regulated learning was also positively related to their studied exemplar test performance, b = .69 (SE = .11), t = 6.10, *p* < .001, CI [.47, .91]; that is, relative to incorrect practice classification trials, when students in the retrieval practice group correctly answered a practice classification trial during self-regulated learning, they were 1.99 times more likely to get that exemplar correct on the studied exemplar test. The remaining predictors were not significant, ts < 1, ps > .37.

### 3.5. Performance on the Novel Exemplar Test

There was no difference in performance on the novel exemplar test between the study group (M = .56, SE = .03) and the retrieval practice group, (M = .55, SE = .02), t < 1, *p* = .73. To evaluate the impact of prior knowledge on students’ test performance for novel exemplars at the item-level (0 = incorrect, 1 = correct), we conducted logistic MEMs with random participant effects. In the first analysis, we included the following predictors: group (0 = study group, 1 = retrieval practice group), change in prior knowledge (group-centered), the total number of trials completed across both phases (group-centered), the proportion of switch trials across both phases (group-centered), the interaction between group and prior knowledge change, the interaction between group and the proportion of switch trials, the interaction between prior knowledge change and the proportion of switch trials, and the three-way interaction between group, prior knowledge change, and the proportion of switch trials. The only significant predictor was the total number of trials completed during self-regulated learning, b = .01 (SE = .004), t = 3.40, *p* = .001, CI [.01, .02]. With each additional trial completed during self-regulated learning, students were 1.01 times more likely to correctly classify a novel exemplar. The remaining predictors were not significant, ts < 1, ps > .34.

We conducted a similar analysis for the retrieval practice group only to explore whether their practice classification performance during self-regulated learning impacted their performance on the novel exemplar test. A logistic MEM was conducted predicting students’ item-level novel test performance (0 = incorrect, 1 = correct) from the following predictors: change in each student’s prior knowledge (group-centered), the total number of trials completed across both phases (group-centered), the proportion of switch trials across both phases (group-centered), the change in their practice classification performance across trials (group-centered), the interaction between prior knowledge change and the proportion of switch trials, the interaction between prior knowledge change and change in practice classification performance, the interaction between the proportion of switch trials and change in practice classification performance, and the three-way interaction between prior knowledge change, the proportion of switch trials, and change in practice classification performance. The only significant predictor was the total number of trials completed during self-regulated learning, b = .02 (SE = .01), t = 2.79, *p* = .005, CI [.01, .03]. With each additional trial completed during self-regulated learning, students were 1.02 times more likely to correctly classify a novel exemplar. The remaining predictors were not significant, ts < 1.80, ps > .07.

### 3.6. Follow-Up Prompts and Questions Regarding Students’ Rationale for Their Study Decisions

Results for each follow-up study decision prompt can be found in Table 3. Most students reported that they selected to switch to a different rock type because they wanted to compare the features of one rock type to the features of a different rock type. Many students also reported that they switched between rock types because they thought it was best to see exemplars of different types back-to-back, and because they felt they knew a rock type well enough and wanted to move on.

Most students reported that they selected to stay within a rock category because they wanted to see which feature the rock type had in common by seeing multiple exemplars. Many students also reported that they stayed within a rock type because they thought it was best to see several exemplars of the same rock type in a row, and because they felt they had not learned the rock type well and wanted to study it again. Chi-square analyses on each prompt revealed that there were no significant differences in students’ endorsements between the study group and the retrieval practice group, χ^2^s ≤ 2.97, ps ≥ .085.

Results for each question assessing students’ beliefs about the role of domain expertise for making study decisions can be found in Table 4. Most students reported that they thought a geology expert would make different study order decisions than would a geology novice (study group, 84.9%, *n* = 28; retrieval practice group, 72.3%, *n* = 24; χ^2^ = 1.45, *p* = .228). Of the students who reported that experts would study differently than would novices, most students reported that geology experts would make many switch study decisions; experts would likely decide to switch more than half of the trials. By contrast, most students reported that geology novices would make many stay study decisions; novices would likely decide to stay on half or more of the trials. Chi-square analyses revealed that, relative to the retrieval practice group, the study group was significantly more likely to report that a novice would often select to switch categories and only stay with a category a few times, χ^2^ = 4.74, *p* = .029. There were no other significant differences between groups, χ^2^s ≤ 2.97, ps ≥ .085.

## 4. Discussion

We explored the degree to which students’ decisions to switch (interleave) during their learning are related to their prior knowledge in the domain and to completing retrieval practice trials during self-regulated learning. Concerning prior knowledge, students’ knowledge in geology and of classifying igneous, metamorphic, and sedimentary rocks increased from Phase 1 to Phase 2 of the experiment. This was true of students’ self-rated knowledge and of objective tests of classification performance, and for students in both groups. Even so, prior knowledge was not related to students’ study order decisions.

These outcomes may indicate that there is no meaningful relationship between students’ decisions to switch (interleave) their study and their level of knowledge about the to-be-learned materials. If so, this is unfortunate given the existing evidence from experimenter-controlled learning contexts that has shown that the study order that benefits learners most can differ for low knowledge students and high knowledge students (Braithwaite and Goldstone 2012; 2015; Rau et al. 2010; but see Osana et al. 2017). However, the lack of a relationship between prior knowledge and study order decisions may be better understood in the context of students’ self-reported beliefs about learning. Most students indicated that a geology expert and novice would make different study decisions. Specifically, a geology expert would often decide to switch between categories whereas a geology novice would often decide to stay within a category. One possibility is that students in the reported experiment considered their level of knowledge in geology to be low during both Phase 1 and Phase 2 despite the gains in knowledge that were observed. In support of this possibility, students’ self-rated level of knowledge increased from Phase 1 to Phase 2, but the Phase 2 averages remained low (i.e., *M*s = 2.33 and 2.64 on the 1–7 scale), and no students gave expert-level ratings (i.e., ratings of 6 or 7) for their geological knowledge.

Students had good reason to provide generally low self-ratings of knowledge while still recognizing the knowledge they gained by the end of the experiment. Students’ practice classification performance in the retrieval practice group did not substantially improve from Phase 1 to Phase 2. Moreover, even though students’ prior knowledge doubled from Phase 1 (16%) to Phase 2 (31%), performance on the Phase 2 prior knowledge test was low, and there is ample room for students to improve on these measures. Most important, low performance on the prior knowledge assessments constrained our ability to detect a relationship between prior knowledge and students’ study order choices (cf. Witherby and Carpenter 2022). These outcomes reveal important directions for future research. It will be valuable to evaluate the relationship between prior knowledge and students’ study order choices when larger gains in prior knowledge are observed. For instance, students in the reported experiment received blocked category instruction in their Understanding the Earth course because they had separate units on each basic-level category (igneous, metamorphic, and sedimentary), and they interleaved practice of the subcategories for the group laboratory assignment associated with each. It is possible that interleaved instruction of the three categories would reveal larger changes in domain knowledge, and thus, students would alter their study order choices. Such investigations are critical for increasing understanding of the factors that impact students’ self-regulated learning and identifying contexts in which students make effective study choices to support their learning of complex concepts.

In contrast with prior knowledge, a relationship was observed between students’ switch decisions and completing retrieval practice trials during self-regulated learning. Students who completed practice classification trials were more likely to switch to a different rock category on the subsequent trial (i.e., to interleave) compared with students who did not complete practice classification trials. Indeed, the switch rate increased by 65% in the retrieval practice group (proportion of switch trials: *M* = .66) relative to the study group (proportion of switch trials: *M* = .40). Thus, engaging in retrieval practice by completing practice classification trials during learning encouraged switch decisions, resulting in increased rates of interleaving. Students likely interpreted successful retrievals during practice classification (i.e., correct practice classification trials) as indicators that that a category of rock was learned well enough to move to a different category. Indeed, students reported that when they decided to switch between categories, they did so to (a) compare features between categories, (b) see different examples, and (c) move to a new category because they thought they knew the current one. Unsuccessful retrievals during practice classification (i.e., incorrect practice classification trials) were likely viewed as evidence that the category was not well learned. Consistent with this proposal, students reported that when they decided to stay within a category, they did so to see (a) consistent features, (b) more of the same examples in a row, and (c) the category again because they did not think they knew it well. Students in the study group reported a similar rationale for their study choices. However, in the absence of practice classification and feedback on performance during self-regulated learning, students in the study group may have experienced difficulty determining their ability to classify each exemplar (cf. Agarwal et al. 2008; Ariel et al. 2021; Little and McDaniel 2014; Tullis et al. 2013). In this case, students may have adopted a heavier stay (blocked study) strategy because they assessed that their knowledge in each category was low.

The finding that retrieval practice during self-regulated learning increased switch decisions is encouraging because, in some instances, interleaved study order has benefitted students’ learning (Eglington and Kang 2017; Kang and Pashler 2012; Kornell and Bjork 2008; Rohrer and Taylor 2007; Samani and Pan 2021; Yan et al. 2016; Zulkiply et al. 2012; Yan and Sana 2021; Yan et al. 2020; for recent meta-analyses see Brunmair and Richter 2019; Firth et al. 2021). However, in the reported experiment, switch rate was not associated with improved performance on either test for students in either the study group or the retrieval practice group. These outcomes are consistent with others that reveal that interleaved study order does not benefit category learning in all contexts (Abel et al. 2021; Carvalho and Goldstone 2014; Elio and Anderson 1981; Lu et al. 2020; Shah et al. 2016; for moderators see Brunmair and Richter 2019; Firth et al. 2021), and that the most effective study order may differ for low and high knowledge students (Braithwaite and Goldstone 2012, 2015; Rau et al. 2010; but see Osana et al. 2017). To provide clear educational recommendations in these multifaceted instances, more research is needed evaluating the impact of retrieval practice during self-regulated learning on students’ study order decisions and performance on final tests of knowledge.

Better performance during practice classification was associated with better performance on the studied exemplar test, which is consistent with the field of research establishing the learning benefits of retrieval practice (Carpenter et al. 2022; Roediger and Karpicke 2006). Even so, it was surprising that group differences did not arise on either the studied exemplar or novel exemplar classification test. Specifically, students who completed practice classification trials during self-regulated learning performed similarly to students who completed study trials on both tests. This does not replicate prior research that found benefits of retrieval practice relative to study conditions on classification performance (Jacoby et al. 2010). There are several reasons why the retrieval practice group did not outperform the study group on the final tests in the reported experiment. We used materials from a different domain relative to the prior research, and category structure can impact the effectiveness of learning strategies (Carvalho and Goldstone 2014; Lu et al. 2020). Furthermore, our students self-regulated their learning by making switch or stay choices, whereas, in Jacoby et al. (2010), students’ learning was experimenter-controlled. This difference may be critical for identifying contexts in which retrieval practice benefits learners, and systematic investigations of retrieval practice during self-regulated category learning will be an important direction for future research.

## 5. Conclusions

We found that changes in students’ domain knowledge were not significantly related to switch decisions during self-regulated learning. However, students reported that they believed domain knowledge would impact study order decisions, indicating that future work should explore how larger increases in domain knowledge may impact study decisions. We also found that students who completed retrieval practice during self-regulated learning made more switch decisions than did students who completed study trials. Interestingly, neither domain knowledge nor retrieval practice were associated with final test performance. Future research should aim to understand the complex relationships between students’ domain knowledge, self-regulated learning, retrieval practice, and final test performance.

## Figures and Tables

**Table 1:** Students’ Reponses to Prior Experience Questions at Phase 1 and Phase 2.

Phase 1 Questions		Study Group	Retrieval Practice Group
1.	Are you currently or have you ever been a Geology Major or Minor?	*n* = 1	*n* = 1
2.	Are you currently or have you ever been an Applied Geoscience Major?	0	0
3.	Do you collect rocks?	*n* = 3	*n* = 3
4.	Do you own a rock and mineral field guide?	0	0
**Phase 2 Questions**			
1.	Do you collect rocks?	*n* = 2	*n* = 4
2.	Do you own a rock and mineral field guide?	*n* = 1	*n* = 1

*Note*. Values represent the number of students who selected “yes”. Questions are listed in the order by which they were presented. Students answered these questions by selecting “yes” or “no” buttons displayed beneath each question.

**Table 2:** Students’ Performance on Coursework Related to the Experiment.

Course Assignment		Study Group	Retrieval Practice Group
**Between Phase 1 and Phase 2**			
1.	Igneous and Metamorphic Rocks Quiz	.94 (.04)	.99 (.01)
2.	Igneous and Metamorphic Rocks Laboratory Assignment	.93 (.01)	.96 (.01) *
3.	Sedimentary Rocks Quiz	.91 (.04)	.98 (.01)
4.	Sedimentary Rocks Laboratory Assignment	.93 (.01)	.93 (.01)
5.	Mid-term Exam	.80 (.02)	.83 (.02)
**After Phase 2**			
6.	Final Exam	.71 (.02)	.74 (.02)

*Note.* Assignments 1 and 3 were quizzes completed individually and online prior to lecture sessions of the course. Each quiz consisted of 6 questions. Assignments 2 and 4 were in-laboratory activities that were completed in small groups. Assignment 5 and 6 were exams completed independently during the lecture portion of the course. Values represent the mean portion of points earned on each assignment. * Significant difference between groups, *p* < .05. Standard error of the mean is indicated in parentheses.

**Table 3:** Students’ Responses about their Study Decisions.

		Overall	Study Group	Retrieval Practice Group
	**Switch study decisions**			
1.	I wanted to compare the features of the previous rock type with the feature of a different rock type.	63.64%	66.67%	60.61%
2.	I thought it was best to see examples from different rock types back-to-back.	51.52%	48.49%	54.55%
3.	I felt like I knew the rock type well enough, and I wanted to move on.	50.00%	39.39%	60.61%
4.	I had seen several of the same rock type in a row and I was tired of looking at that rock type.	31.82%	33.33%	30.30%
5.	I thought the previous rock type was too challenging and I wanted to practice something else.	9.10%	15.15%	3.03%
6.	I never selected to study a different type of rock when I correctly answered a practice trial.	0%	0%	0%
	**Stay study decisions**			
7.	I wanted to see which features the rock type had in common by seeing multiple examples from the category.	75.76%	69.70%	81.82%
8.	I thought it was best to see several of the same rock type in a row.	68.18%	63.63%	72.73%
9.	I didn’t think I knew the rock type well enough and wanted to practice it again.	57.58%	48.49%	66.67%
10.	I wanted to verify that I really knew the rock type before I moved on.	50.00%	54.55%	45.45%
11.	I wanted a reminder of the correct rock type that I just studied.	37.88%	42.42%	33.33%
12.	I had not seen this rock type before, and I wanted to see more examples.	19.70%	21.21%	18.18%
13.	I never selected to study the same type of rock when I incorrectly answered a practice trial.	3.03%	6.06%	0%

*Note*. Switch study decisions refers to students’ responses to, “When I selected to study a different type of rock, I did so because”. Stay study decisions refers to students’ responses to, “When I selected to study the same rock, I did so because”. Responses are listed from most endorsed to least endorsed (overall). The percent of students who endorsed each response are provided. Students could select as many responses as desired for each question, so the total percent of students exceeds 100 for each question.

**Table 4:** Students’ Responses of their Beliefs about the Impact of Expertise on Study Decisions.

	Overall	Study Group	Retrieval Practice Group
**Beliefs about the study choices of a novice**			
Always select to study the same type of rock.	17.31%	17.86%	16.67%
Often select to study the same type of rock but also select to study a different type of rock a few times.	36.54%	28.57%	45.83%
Select to study the same type of rock half of the time and a different type of rock for the other half of the time.	30.77%	28.57%	33.33%
Often select to study a different type of rock but also select to study the same type of rock a few times.	9.62%	17.86%	0% *
Always select to study a different type of rock.	5.77%	7.14%	4.17%
**Beliefs about the study choices of an expert**			
Always select to study the same type of rock.	3.85%	7.14%	0%
Often select to study the same type of rock but also select to study a different type of rock a few times.	11.54%	10.71%	12.50%
Select to study the same type of rock half of the time and a different type of rock for the other half of the time.	7.69%	14.29%	0%
Often select to study a different type of rock but also select to study the same type of rock a few times.	32.69%	35.71%	29.17%
Always select to study a different type of rock.	44.23%	32.14%	58.33%

*Note*. The percentage of students who selected each response are provided. The percentage of students was calculated out of the number of students who reported they believed that expert and novices would make different study decisions (*n* = 52). Responses are listed from most staying behavior to the least staying behavior (i.e., all switching). Significant difference in response rate between the study group and the retrieval practice group, * *p* = .03.

## Data Availability

All materials and raw data have been uploaded to the Open Science Framework and can be accessed at https://osf.io/za328/?view_only=52e47042b66c4b4cb151dd7d99e9f57e. The preregistration can be accessed at https://doi.org/10.17605/OSF.IO/UQWZS.
